# Perfluoropolyether Nanoemulsion Encapsulating Chlorin e6 for Sonodynamic and Photodynamic Therapy of Hypoxic Tumor

**DOI:** 10.3390/nano10102058

**Published:** 2020-10-19

**Authors:** Liang Hong, Artem M. Pliss, Ye Zhan, Wenhan Zheng, Jun Xia, Liwei Liu, Junle Qu, Paras N. Prasad

**Affiliations:** 1 Key Laboratory of Optoelectronic Devices and Systems of Ministry of Education and Guangdong Province, College of Optoelectronic Engineering, Shenzhen University, Shenzhen 518060, China; hongliang0702@126.com; 2 Institute for Lasers, Photonics and Biophotonics, University at Buffalo, State University of New York, Buffalo, New York, NY 14260, USA; ampliss@buffalo.edu; 3 Department of Biomedical Engineering, University at Buffalo, State University of New York, Buffalo, New York, NY 14260, USA; yezhan@buffalo.edu (Y.Z.); wzheng26@buffalo.edu (W.Z.); junxia@buffalo.edu (J.X.)

**Keywords:** photodynamic, sonodynamic, hypoxia, nanoemulsion, delivery, tumor

## Abstract

Sonodynamic therapy (SDT) has emerged as an important modality for cancer treatment. SDT utilizes ultrasound excitation, which overcomes the limitations of light penetration in deep tumors, as encountered by photodynamic therapy (PDT) which uses optical excitations. A comparative study of these modalities using the same sensitizer drug can provide an assessment of their effects. However, the efficiency of SDT and PDT is low in a hypoxic tumor environment, which limits their applications. In this study, we report a hierarchical nanoformulation which contains a Food and Drug Administration (FDA) approved sensitizer chlorin, e6, and a uniquely stable high loading capacity oxygen carrier, perfluoropolyether. This oxygen carrier possesses no measurable cytotoxicity. It delivers oxygen to overcome hypoxia, and at the same time, boosts the efficiency of both SDT and PDT. Moreover, we comparatively analyzed the efficiency of SDT and PDT for tumor treatment throughout the depth of the tissue. Our study demonstrates that the strengths of PDT and SDT could be combined into a single multifunctional nanoplatform, which works well in the hypoxia environment and overcomes the limitations of each modality. The combination of deep tissue penetration by ultrasound and high spatial activation by light for selective treatment of single cells will significantly enhance the scope for therapeutic applications.

## 1. Introduction

**Scheme 1:**
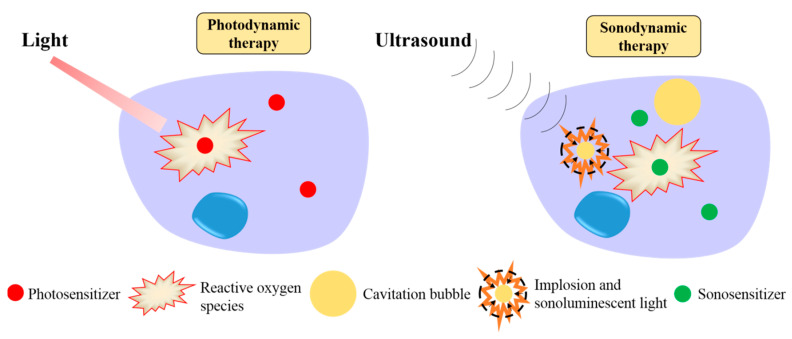
Mechanisms of photodynamic therapy and sonodynamic therapy.

Two closely related cancer therapeutic approaches, photodynamic therapy (PDT) and sonodynamic therapy (SDT), offer tumor destruction with unsurpassed anatomical precision. In these approaches, a therapeutic agent (photosensitizer in PDT and sonosensitizer in SDT) is delivered to the tumor. Then, in response to excitation with either light or ultrasound for PDT and SDT, respectively, the photo- or the sono-sensitizer generates highly toxic reactive oxygen species (ROS), which destroy the tumor cells (Scheme 1) [1,2]. The advantages of PDT and SDT include noninvasiveness, unsurpassed targeting, and low side effects in untreated areas [3,4].

At the same time, the scope of PDT and SDT applications at present is inherently narrow. First, the generation of ROS by either PDT or SDT for most sensitizers relies on the presence of O_2_ in the medium. However, tumors are commonly hypoxic, which makes ROS generation ineffective [5,6]. Hypoxia is a characteristic hallmark of the microenvironment in solid tumors, resulting on one side from upregulated metabolism, especially in fast-growing aggressive tumors, and on the other side, from insufficient oxygenation through aberrant blood vessels [7,8,9]. Moreover, the conversion of O_2_ to ROS throughout PDT and SDT rapidly depletes available oxygen, which constrains the treatment duration [6,10]. It has to be emphasized that hypoxia not only strongly correlates with the aggressiveness of cancer, but also promotes tumor metastasis and tumor resistance to chemotherapy, radiotherapy, and immunotherapy [11,12,13]. Therefore, overcoming the hypoxic limitation in PDT and SDT is critical for advancing the efficiency of these techniques [6,14,15,16].

Another challenging problem in both PDT and SDT is effective excitation of photo- and sono-sensitizers in remote tumors. In the case of PDT, poor penetration of light through biological tissues, even in the near-infrared (NIR) range, limits the treatment to only superficial or endoscopically accessible tumors [17]. Moreover, it is difficult to treat large tumors, even when they are easily accessible [18]. To enhance light penetration through biological tissues, two-photon excitation of PDT drugs with NIR light in the biological transparency window has been proposed [19,20,21]. An additional benefit of two-photon PDT is the highly localized excitation, which mitigates damages to healthy surrounding tissues [22]. This feature is highly demanded in the treatment of tumors in brain and other vital organs [23]. However, two-photon PDT relies on high light irradiation intensities due to the intrinsically low two-photon cross-sections of PDT drugs [24], which reduces the efficiency of ROS generation and may produce severe photothermal damage of untargeted tissues surrounding the tumor.

In comparison with PDT, SDT has its distinct strengths and limitations. Ultrasound can penetrate deeply through the tissue, reaching tumors at depths of up to 10 cm [25,26,27,28]. The high penetration depth allows SDT to treat essentially any tumor in the human body. However, the spatial precision of SDT is inherently lower than that of PDT. The spatial specificity of non-focused ultrasound is within a few centimeters range. Even using specially designed transducers, the focus of ultrasound is still several cubic millimeters [28,29,30]. The poor spatial precision makes it tricky to avoid unintended damage of healthy tissues proximal to or interspacing the tumors [26,31], and consequently, limits the application scope of SDT. For example, glioma brain tumors infiltrate healthy brain tissues deeply in the skull, which renders SDT ineffective for selective eradication of all tumor reservoirs [32,33]. In comparison, light has inherently higher spatial selectivity than sound. For instance, two-photon excitation approaches submicron resolution in 3D, which highlights the merits of PDT for treatment of brain tumors [34,35,36]. In conclusion, treatment of deep-seated and highly diffusive tumors like glioma may require a creative integration of light and sound technologies.

Although the potential of combining PDT and SDT has been reported in several pioneering studies [26,31,37,38,39,40], these studies did not analyze the relative merits and shortcomings of PDT/SDT approaches for treatment of the same tumor.

In parallel, to enhance the efficiency of PDT or SDT, hypoxia-overcoming nanoformulations have been designed [16,41,42,43,44,45]. Recent approaches include oxygenation by a hydrogen peroxide (H_2_O_2_) catalysis [46,47] and oxygen delivery using hemoglobin [48,49]. While these early studies have shown a significant potential of oxygenation for PDT and SDT, the efficiency of currently available nanoformulations is not sufficiently high, due to the limited amount of H_2_O_2_ in the tumor microenvironment and the inadequate oxygen loading capacity of hemoglobin [50]. To overcome this limitation, highly efficient perfluorocarbon (perfluorobutane, perfluoropentane, perfluorohexane, etc.)-based carriers have been tested [43]. The oxygen loading efficiency of perfluorocarbons is intrinsically higher than that of hemoglobin-based formulations. At 25 °C under 1 atm, 100 mL of perfluorocarbon carries about 40–50 mL O_2_, while the same volume of oxygenated blood carries only ~20 mL O_2_ [50,51]. However, perfluorocarbon-based compounds are not stable and will spontaneously transit to gas at room temperature, which causes a loss of perfluorocarbons during the preparation process and may also hamper the treatment efficiency after perfluorocarbons reach the tumor [52].

To date, we still miss a clear concept of how to combine PDT and SDT for multidimensional treatment of tumors throughout the human body. This unified concept, operating with an effective ROS-synthesizing drug, has to meet the following requirements to achieve reproducible eradication of tumors, while minimizing the side effects: (1) variable depth of penetration in tissues by each modality, (2) high spatial precision in drugs excitation, (3) amplification of treatment efficiency by the combination of ultrasound and light excitation, and (4) controlled alleviation of hypoxia.

In our study, we introduce an integrated nanoplatform towards unified implementation of PDT and SDT under hypoxia. Herein, we developed a multifunctional nanoformulation, chlorin e6-perfluoropolyether in water nanoemulsion (Ce6-P/W NE). Our nanoformulation includes a Food and Drug Administration (FDA)-approved sensitizer that can be used for both PDT and SDT, and a novel perfluoropolyether (PFPE)-based oxygen carrier with unparalleled stability to reduce hypoxia and boost the efficiency of ROS generation. With a promising integration of high 3D precision of photoexcitation, deep penetration of ultrasound, and effective generation of ROS boosted by oxygen delivery, this unified nanoplatform presents an intriguing avenue for expanding the capabilities of PDT and SDT.

## 2. Materials and Methods

### 2.1. Materials

PFPE (Fluorolink^®^ MD700) was obtained from Solvay (Brussels, Belgium). Pluronic (Mn 2900) and dimethyl sulfoxide (DMSO) were provided by Aladdin Industrial Corporation (Shanghai, China). Chlorin e6 (Ce6) was purchased from Frontier Scientific (Logan, UT, USA). 1,3-diphenylisobenzofuran (DPBF) was purchased from TCI (Tokyo, Japan). PC-3 cells (prostate cancer cells) were obtained from American Type Culture Collection (ATCC, Manassas, VA, USA). Advanced Dulbecco’s modified Eagle’s medium (DMEM) medium was provided by Thermo Fisher Scientific (Bremen, Germany). Cell Counting Kit-8 (CCK-8) was purchased from Dojindo Laboratories (Kumamoto, Japan). Double distilled water was used throughout the experiments.

### 2.2. Preparation of Ce6-P/W NE

A mixture of 0.006 g Ce6, 0.21 g Pluronic (Mn 2900) and 0.09 g PFPE was stirred at 500 rpm for 10 min, and then, bath sonicated for 30 min. Afterwards, 2.7 mL water was titrated to the mixture under stirring at 500 rpm, and the stirring was continued for another hour after the titration. The resultant dispersion was filtered three times with a 0.45-μm syringe filter (Millipore, Burlington, NJ, USA) and then, once with a 0.2-μm syringe filter (Millipore, Burlington, NJ, USA). The obtained Ce6-P/W NE contains nanodroplets dispersed in water. The Ce6 loading efficiency and entrapment efficiency were calculated to be 1.95% and 97.63%, respectively.

### 2.3. Characterization

The optical absorption spectra of Ce6-P/W NEs and P/W NEs were recorded using a UV–vis–NIR scanning spectrophotometer (UV-3101PC, Shimadzu, Kyoto, Japan). The droplet size distribution and zeta potentials of Ce6-P/W NEs were measured by a dynamic light scattering technique using a 90 Plus Particle Size Analyzer (Brookhaven Instruments Corporation, Holtsville, NY, USA) at 25 °C. The Ce6-P/W NEs were diluted 1:120 in water for the measurements. The refractive index used was 1.33. To investigate the storage stability, the undiluted Ce6-P/W NEs (water dispersions rather than by dissolving freeze-dried powders) were kept still and stored at 4 °C, and the droplet size was determined every 10 days. The appearance of Ce6-P/W NEs was observed before the droplet size measurements. Fluorescence emission spectra were acquired using 10 mm path length cuvettes with a slit width of 1 nm at 298 K on a Fluoromax-4 spectrofluorometer (Horiba, Fukuoka, Japan). To evaluate its physical stability in the physiological environment, Ce6-P/W NEs were diluted 1:120 in DMEM culture medium supplemented with 10% fetal bovine serum or in phosphate-buffered saline (PBS), and the mean droplet diameters were determined after storage at 37 °C for 0, 2, 4, 8, 12, and 24 h.

### 2.4. Singlet Oxygen (^1^O_2_) Production in Response to Ultrasound or Light

^1^O_2_ production was quantitatively analyzed using the ^1^O_2_-specific probe DPBF. All procedures were performed in the dark. DPBF was thoroughly dissolved in DMSO to obtain a 10 mg/mL solution, and then, 10 μL of DPBF solution was added to 1.5 mL water-diluted Ce6-P/W NE, Ce6 emulsion (Ce6 E, containing equal amount of Ce6 as Ce6-P/W NE), or water. Ce6 E was prepared following the same procedure as Ce6-P/W NE, except that PFPE was absent and the filtration was performed and was filtered two times with a 0.45-μm syringe filter (Millipore, Burlington, NJ, USA). For tests, Ce6 E was diluted until it had the same Ce6 concentration as Ce6-P/W NE. The content of Ce6 was evaluated by absorption at 404 nm. To create a hypoxic condition, the samples were bubbled with a positive pressure of Ar gas for 5 min. Afterwards, ultrasound (0.25 W/cm^2^, 2.1 MHz, 1 min) irradiation or laser (633 nm, 50 mW/cm^2^, 2 min) irradiation was applied. The absorption values at 424 nm before (A_0_) and after (A_1_) irradiation were recorded and normalized (100 × (A_0_ − A_1_)/A_0_) to quantify the production of ^1^O_2_ [41,53]. To perform sonication, a piezo hemisphere ultrasound transducer (resonant frequency 2.1 MHz, dimensions: 20 mm diameter × 30 mm curvature (radius), Steiner and Martins, Inc., Davenport, FL, USA) with a focal length of 30 mm was placed at the bottom of a water bath. The samples were placed at the focal area of the transducer for insonation.

### 2.5. Cell Culture

PC-3 cells or Hela cells were cultured with an advanced DMEM medium supplemented with 3% fetal bovine serum and 1% streptomycin/penicillin at 37 °C in a humidified atmosphere with 5% CO_2_.

### 2.6. SDT Efficacy

SDT efficacy was investigated using cell viability assays. PC-3 cells were seeded into 96-well plates at a density of 8000 cells/well, and allowed to grow for 24 h. Ce6-P/W NEs were diluted with a serum-free advanced DMEM medium to obtain different concentrations (200 and 400 μg/mL, calculated as the weight of the entire nanodroplet including Pluronic Mn 2900 and PFPE divided by total volume). The medium was changed for Ce6-P/W NEs, Ce6 Es (with equal Ce6 content as Ce6-P/W NE), or fresh serum-free medium, and the cells were incubated at room temperature for 40 min. Afterwards, Ce6-P/W NEs, Ce6 Es, and serum-free medium in the 96-well plates were withdrawn, and fresh serum-free medium was added into the 96-well plates. Then, the cells were treated with or without sonication (0.25 W/cm^2^, 2.1 MHz, 1 min) as described in the section “2.4 Singlet Oxygen (^1^O_2_) Production in Response to Ultrasound or Light”. Subsequently, the cells were incubated at 37 °C, 5% CO_2_ for 24 h. The medium was changed with a mixture of 10 μL CCK-8 and 90 μL serum-free medium. After incubation at 37 °C, 5% CO_2_ for 1 h, the absorbance at 450 nm was measured using a Dynex Opsys MR™ Microplate Reader (Aspect Scientific Ltd., Cheshire, UK).

### 2.7. PDT Efficacy

PDT efficacy was examined by Calcein AM and propidium iodide (PI) staining and imaging with confocal laser scanning microscopy. Experiments were performed in the dark. PC-3 cells (for irradiation in a normoxic condition) or Hela cells (for irradiation in a hypoxic condition) were placed into Petri dishes with optical-glass bottom windows (Cellvis, Mountain View, CA, USA) and then, incubated at 37 °C, 5% CO_2_ for 24 h. Afterwards, cells were incubated with 400 μg/mL Ce6-P/W NEs or Ce6 Es containing equal amounts of Ce6 at 37 °C for 2 h. The cells were washed 3 times with culture medium, and then, irradiated with a laser (633 nm, 50 mW/cm^2^) for 30 or 45 s in a normoxic condition and 105 s in a hypoxic condition. The laser was perpendicular to the Petri dish, and the distance between the laser source and Petri dish was 50 mm. The hypoxic condition was created using AnaeroPouch^®^ Anaero (Mitsubishi Gas Chemical Co., Tokyo, Japan) following the manufacturer’s instructions. After laser irradiation, the cells were incubated with the advanced DMEM medium supplemented with 3% fetal bovine serum at 37 °C for 2 h, and then, incubated for 40 min in a serum-free culture medium containing 1 µmol/L of Calcein AM (excitation/emission peaks are at 495/515 nm) and 500 nmol/L of PI (excitation/emission peaks are at 536/617 nm). Subsequently, the cells were washed 3 times and then, imaged with an SP2 confocal laser scanning microscope (Leica, Wetzlar, Germany). The PI fluorescence intensities were analyzed using Image J 1.52v software.

### 2.8. Cytocompatibility

To investigate the cytocompatibility of Ce6-P/W NEs, PC-3 cells were seeded in 96-well plates at a density of 8000 cells/well and incubated at 37 °C, 5% CO_2_ for 24 h. Afterwards, the cells were incubated in the dark with serum-free medium or various concentrations of Ce6-P/W NEs at 37 °C, 5% CO_2_ for 24 h. Then, the medium and Ce6-P/W NEs were replaced with the mixture of 10 μL CCK-8 and 90 μL serum-free medium and incubated for 1 h. Finally, the absorbance at 450 nm was recorded using a Dynex Opsys MR™ Microplate Reader (Aspect Scientific Ltd., Cheshire, UK).

### 2.9. Comparison between SDT and PDT on Tissue Penetration

**Scheme 2:**
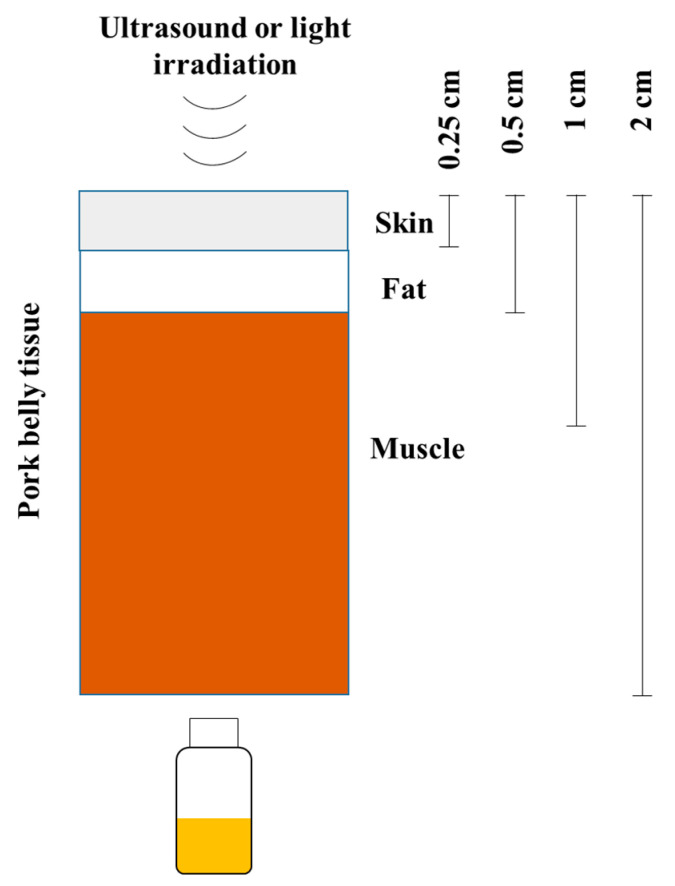
Procedure for singlet oxygen production comparison between sonodynamic therapy and photodynamic therapy.

To compare the penetrating ability of SDT and PDT, ^1^O_2_ production of Ce6-P/W NEs was measured after ultrasound (0.25 W/cm^2^, 2.1 MHz, 1 min) or white light (60 lumens, LED flashlight, Rayovac, Middleton, WI, USA, 1 min) was applied on the top of pork belly tissues with depth of 0, 0.25 (skin), 0.5 (skin and fat), 1 (skin, fat, and muscle), or 2 (skin, fat, and muscle) cm (Scheme 2). The ^1^O_2_ production of Ce6-P/W NEs was determined as stated above, with samples placed facing the underside of the pork belly tissue in the normoxic condition.

## 3. Results and Discussion

### 3.1. Preparation and Characterization of an Oxygen-Carrying Photo-Sonotherapeutic (Ce6-P/W NE)

**Scheme 3:**
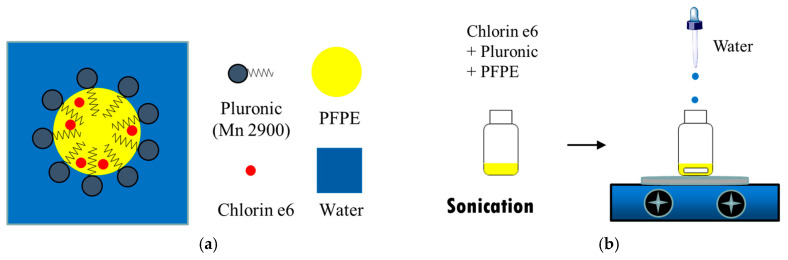
Schematic representation of structure (**a**) and preparation method (**b**) of Ce6-P/W NE. Ce6-P/W NE, chlorin e6-perfluoropolyether (PFPE)/water nanoemulsion.

The biomedical application of PFPE is limited by its immiscibility with aqueous solutions [54]. To address this limitation, we created a new nanoemulsion (NE) type, PFPE in water (P/W) (Scheme 3a), via the phase inversion composition (PIC) method (Scheme 3b) [55]. The P/W NE consists of nanodroplets dispersed in water. The nanodroplet is composed of an emulsifier layer and a PFPE core. The core was loaded with hydrophobic sensitizer, Ce6. An added benefit of this nanoformulation is that it facilitates the aqueous solubility of hydrophobic Ce6, a prerequisite for injection of Ce6 into the human body. It is worth noting that besides acting as a photo- and sono-sensitizer, Ce6 also possesses the abilities of photoacoustic and fluorescence imaging [56], allowing for theranostic applications.

**Figure 1:**
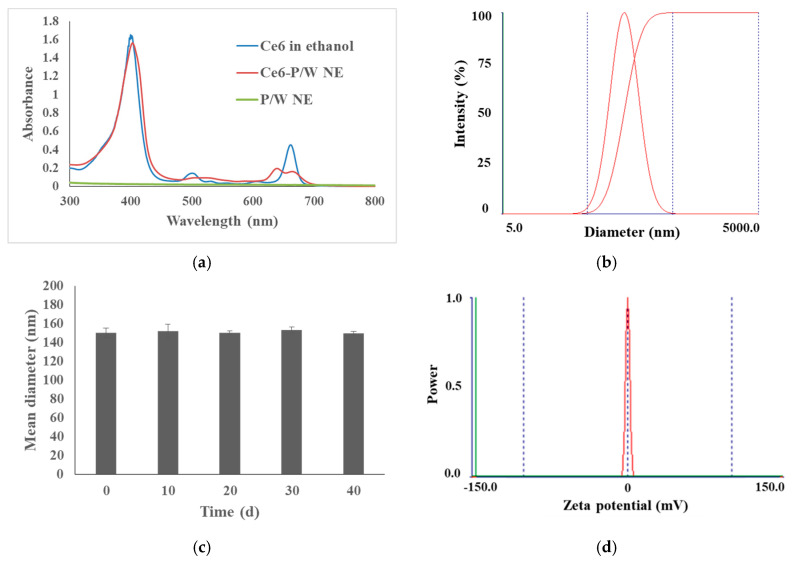

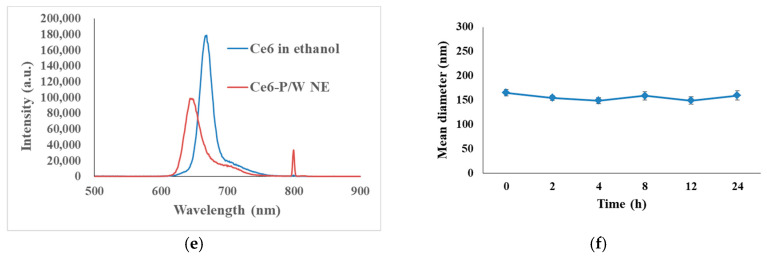
Characterization of physicochemical properties of Ce6-P/W NE nanodroplets. (**a**) Ultraviolet–visible light absorption spectrum. Ce6 concentration, 12 μg/mL. (**b**) Hydrodynamic diameter. Ce6 concentration, 16 μg/mL. (**c**) Physical stability: Plot shows droplets size for different storage periods at 4 °C. Ce6 concentration, 16 μg/mL. (**d**) Zeta potential. Ce6 concentration, 16 μg/mL. (**e**) Fluorescence emission spectra of Ce6 dissolved in ethanol and Ce6-P/W NE with the same Ce6 concentration (12 μg/mL). (**f**) Droplet diameter change of the Ce6-P/W NEs in DMEM cell culture medium supplemented with 10% fetal bovine serum throughout storage at 37 °C for 24 h. Ce6 concentration, 16 μg/mL. All data were presented as mean value ± standard deviation. After Levene’s test for equality of variances, one-way analysis of variance (ANOVA) and Bonferroni multiple comparison test was employed for the comparison of mean values. n = 3. Ce6-P/W NE, chlorin e6-perfluoropolyether (PFPE)/water nanoemulsion.

The absorption spectrum indicates efficient encapsulation of Ce6. The characteristic porphyrins absorption peaks at 404 and 640 nm were present in the Ce6-P/W NE absorption spectrum, while the unloaded P/W NE did not absorb in this spectral region (Figure 1a). The different absorbance characteristics between Ce6-P/W NE and free Ce6 in ethanol may be ascribed to the interactions between the nanoemulsion components and Ce6 (Figure 1a) [43]. The preparation yielded nanostructures with an average diameter of 150.1 ± 5.1 nm and a low polydispersity index (0.155 ± 0.009), pointing to a uniform size of the nanodroplets (Figure 1b). In prospective in vivo applications, the relatively small size and high uniformity are likely to facilitate effective accumulation of Ce6-P/W NEs into the tumor site by passive targeting through the enhanced permeability and retention (EPR) effect [43]. Furthermore, the obtained nanodroplets were stable and suitable for prolonged storage. In particular, we observed neither precipitation nor change of droplet diameter following 40 days of storage at 4 °C (Figure 1c). Electrostatic repulsion and steric repulsion among the droplets coated by emulsifier molecules are two major mechanisms underlying the storage stability of a nanoemulsion system [57,58]. In the Ce6-P/W NE system, on the one hand, Pluronic is a non-ionic emulsifier and therefore, barely generates any electrostatic repulsion, as revealed by the negligible droplet electrical charge (zeta potential −0.02 ± 0.03 mV, Figure 1d). On the other hand, the large hydrophilic polyoxyethylene head-groups of Pluronic create strong and long-range steric repulsion, which prevents the droplets from aggregation [59,60]. To summarize, steric repulsion is likely the dominant mechanism underlying the storage stability of Ce6-P/W NE. Consistent with this research, Pluronic was reported to stabilize nanoemulsion systems encapsulating cyclosporine (or prednisolone) via steric repulsion instead of electrostatic repulsion, with zeta potential results near to zero [61]. The fluorescence emission spectra of Ce6-P/W NE and Ce6 in ethanol further suggest interactions between Ce6 and the nanoemulsion carrier and encapsulation of Ce6 (Figure 1e). To test the stability of Ce6-P/W NE in the physiological environment, Ce6-P/W NE in DMEM culture medium supplemented with 10% fetal bovine serum (Figure 1f) or in PBS (Figure S1) was stored at 37 °C for 24 h. Throughout the storage, neither aggregation nor alteration of droplet size could be detected (*p* > 0.05), which indicated the satisfactory stability of Ce6-P/W NE in physiological solutions.

### 3.2. ^1^O_2_ Generation in Response to Ultrasound and Light Excitation

ROS-mediated destruction of tumor cells is a key mechanism for PDT, SDT, and radiotherapy [62,63]. However, as discussed above, ROS generation is inherently ineffective in the hypoxic regions of tumors [64].

To quantify the effect of hypoxia on ROS generation and to define the efficiency of the oxygen carrier proposed here to boost both PDT and SDT, we performed a series of in vitro measurements. In these experiments, the same concentrations of Ce6, either in the form of an aqueous emulsion (Ce6 Es) or encapsulated in oxygen-carrying nanodroplets (Ce6-P/W NEs), were diluted and bubbled with a positive pressure of Ar gas to create a hypoxic condition.

**Figure 2:**
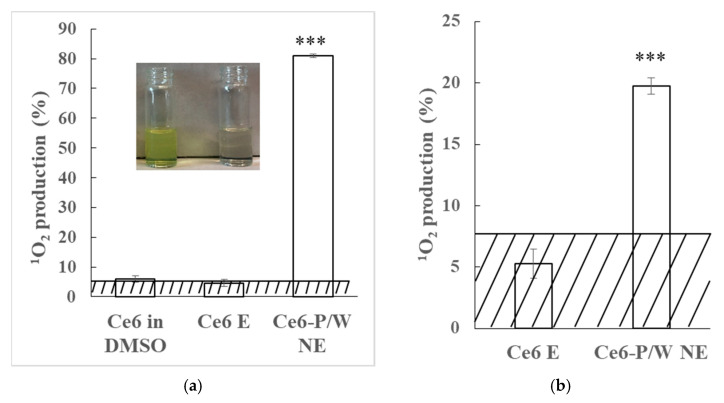
^1^O_2_ production of the Ce6 in DMSO, Ce6 Es, and Ce6-P/W NEs under (**a**) ultrasound (0.25 W/cm^2^, 2.1 MHz, 1 min) or (**b**) light (633 nm, 50 mW/cm^2^, 2 min) irradiation in hypoxic condition at 1 mg/mL Ce6-P/W NE. The concentration was calculated as weight of Pluronic Mn 2900 and PFPE divided by the total volume. Ce6 concentration, 20 μg/mL. The inset is a photo of the Ce6 E (left) and the Ce6-P/W NE (right) with 1,3-diphenylisobenzofuran (DPBF) after ultrasound irradiation. Data were presented as mean value ± standard deviation. After Levene’s test for equality of variances, one-way analysis of variance (ANOVA) and Bonferroni multiple comparison test was employed for the comparison of mean values. n = 3. ***, *p* < 0.001. The shadow represents background signals detected by control (i.e., water containing only DPBF) groups (3.3098 ± 0.9599% for ultrasonic irradiation, and 7.5879 ± 2.1061% for light irradiation). ^1^O_2_, singlet oxygen. DMSO, dimethyl sulfoxide. Ce6 E, chlorin e6 emulsion. Ce6-P/W NE, chlorin e6-perfluoropolyether (PFPE)/water nanoemulsion.

Next, the Ce6 solutions were exposed to either ultrasound or a 633 nm light source, and ^1^O_2_ generation was monitored using DPBF. DPBF is a ^1^O_2_-specific probe whose absorbance peak intensity decreases as it reacts with ^1^O_2_. The more ^1^O_2_ produced, the more absorbance decrease will be detected. Therefore, we used the percentage of absorbance decrease to evaluate ^1^O_2_ production. We observed that under hypoxic conditions, the generation of ^1^O_2_ by Ce6 E was suppressed nearly to the background level under either excitation modality (Figure 2). In striking contrast, we found very significant levels of ^1^O_2_ production in hypoxic solutions by using our PFPE-based nanoformulation under both ultrasound and light excitations. Specifically, the signal of ^1^O_2_ production by Ce6-P/W NE was ~17 times higher under ultrasound excitation (Figure 2a) and ~3 times higher under light excitation (Figure 2b) in comparison with the Ce6 E level. The ^1^O_2_ production by Ce6-P/W NE was so robust that the sample turned from yellow to white, in contrast to the yellow color of the Ce6 E sample after ultrasound irradiation (Figure 2a inset). The ^1^O_2_ production efficiency of Ce6-P/W NE was also remarkably higher than Ce6 in organic solvent DMSO (Figure 2a). Furthermore, the oxygen-carrier nanoformulation provided an effective boost in ROS generation, even under the normal oxygen levels. We registered a ~5 times higher signal of ^1^O_2_ production by the Ce6-P/W NE compared with the Ce6 E in normoxic conditions under ultrasound excitation (Figure S2a).

Thus, while the enhancement of ^1^O_2_ production may vary depending on the concentration of Ce6, excitation power, time of irradiation, temperature, and other variables (Figure S2b), our results indicate a very significant increase in ROS generation by the PFPE oxygen carrier for both SDT and PDT (Figure 2a,b). The efficient oxygen delivery originates from the extraordinary affinity of PFPE to oxygen due to the high electronegativity of fluorine [65]. We conclude that Ce6-P/W NE enhances the effect of SDT and PDT. Besides, it has been widely demonstrated in previous studies that if ultrasound and light are employed simultaneously, ^1^O_2_ generation efficiency and therapeutic efficacy could be enhanced [66,67]. The suitability of Ce6 for both ultrasound and photoexcitation enables the unification of SDT and PDT modalities without increasing the drug exposure dose, thus reducing side effects commonly found in combinatory drug treatments [18,68,69].

### 3.3. Enhanced SDT Efficacy

In the next series of experiments, we modeled SDT in cultured cells. As described in the “Materials and Methods” section, PC-3 prostate cancer cells were incubated with either Ce6 Es or Ce6-P/W NEs (the same Ce6 concentration). Following ultrasound treatment, cellular viability was estimated using the CCK-8 assay. In the control, cells were not treated with Ce6 or not exposed to ultrasound.

**Figure 3:**
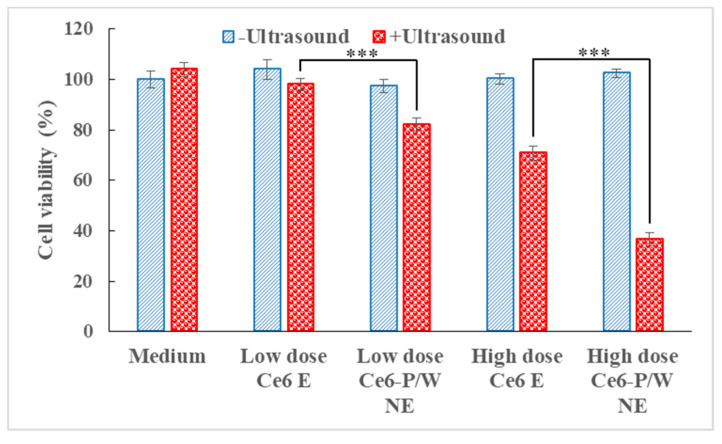
Cell viabilities after incubation with the culture medium, Ce6 Es, or Ce6-P/W NEs with/without ultrasound irradiation (0.25 W/cm^2^, 2.1 MHz, 1 min). Data were presented as mean value ± standard deviation. After Levene’s test for equality of variances, one-way analysis of variance (ANOVA) and Bonferroni multiple comparison test was employed for the comparison of mean values. n = 3. ***, *p* < 0.001. “Low dose” and “high dose” refer to Ce6-P/W NE concentrations (calculated as the weight of the entire nanodroplet including Pluronic Mn 2900 and PFPE divided by total volume) at 200 μg/mL (Ce6 concentration, 4 μg/mL) and 400 μg/mL (Ce6 concentration, 8 μg/mL), respectively. The Ce6 Es contain equal amount of chlorin e6 as Ce6-P/W NEs. Ce6 E, chlorin e6 emulsion. Ce6-P/W NE, chlorin e6-perfluoropolyether (PFPE)/water nanoemulsion.

Notably, a low or negligible level of cytotoxicity in the absence of excitation is the most basic requirement for pharmaceutical photo- and sono-sensitizers [22]. Consistent with that, the control experiments in PC-3 cells revealed no measurable “dark” toxicity of Ce6-P/W NEs in absence of sono- or photo-activation (Figure 3).

In contrast, upon exposure to ultrasound, the formulations reduced the cellular viability. As expected, the cytotoxicity was dependent on the concentration. Under our experimental conditions, we found that sonoexcitation of Ce6 Es reduced little cellular viability (*p* > 0.05 in comparison with the Medium + Ultrasound group), when the Ce6 E was present at a low dose. At a high dose, the cellular viability was reduced by ~33%. In comparison, the Ce6-P/W NE formulation was obviously more effective. At a low dose and a high dose, Ce6-P/W NE decreased cellular viability upon sonoexcitation by 21.8% and 63.4%, respectively. Ce6-P/W NE enables the boosting of SDT efficiency via ROS generation improvement, owing to efficient oxygen delivery by PFPE.

It is noteworthy that the Ce6-P/W NE was able to devastate cancer cells at an ultrasound power density as low as 0.25 W/cm^2^ and ultrasound dose as small as 15 J/cm^2^. In contrast, much higher ultrasound power density and dose were required for other nanoformulations that used Ce6 as the sonosensitizer, yet employing alternative strategies such as H_2_O_2_ decomposition [70] (1 W/cm^2^, 180 J/cm^2^, under normoxia) to address tumor hypoxia. This suggests the high sonodynamic efficiency of Ce6-P/W NE caused by efficient oxygen delivery by PFPE. The experiments were performed in a normoxic rather than hypoxic condition due to difficulties in coupling the ultrasound transducer, hypoxic bag, and the 96-well plate. However, it is highly expected that in the hypoxic condition, Ce6-P/W NE could more significantly augment the SDT efficiency, since a ~17 times higher ^1^O_2_ generation than Ce6 E in hypoxic condition was detected in this research, as shown in Figure 2a.

In addition, ultrasound has the ability to remarkably promote the release of oxygen to the tumor site from perfluorocarbon-based nanostructures like Ce6-P/W NE, as is intensively reported in previous studies [41,71]. The released oxygen contributes to the modulation of tumor hypoxia. Ultrasound facilitates oxygen release by cleaving the affinity between oxygen and F atoms [54].

### 3.4. Enhanced PDT Efficacy

Inspired by the advantage of Ce6-P/W NE over Ce6 E on ^1^O_2_ generation in response to light excitation, we investigated the ability of the two formulations to kill cancer cells under 633 nm laser irradiation in both normoxic and hypoxic conditions. Fluorescence images of Calcein acetoxymethyl ester (Calcein AM)- and PI-stained tumor cells, were used to demonstrate the effect of tumor cell ablation. The intensity of Calcein staining directly correlates with cellular viability. PI is a marker of necrotic cells, since PI could not enter live cells, but could bind to the DNA of necrotic cells and produce fluorescence signals. It should be noted that although the ultrasound used in this research is focused, the irradiation area it can target is still quite broad (at least larger than 32 mm^2^), and all the cells in each well of the 96-well plate were irradiated by ultrasound. This reflects the intrinsically low spatial precision of ultrasound. Therefore, we used the CCK-8 cell viability assay method to detect the viability of all the cells in each well to evaluate SDT efficacy. In contrast, light has remarkably higher spatial precision than ultrasound. The irradiation area of the 633 nm laser in this research is less than 7.1 mm^2^. Hence, we employed Calcein and PI staining to intuitively and clearly show the cell damage caused by light irradiation in comparison to non-irradiated areas.

First, we established a minimum irradiation dose to cause the cytotoxicity in the treated cells. We found that under the normoxic condition, 30 s periods of laser irradiation of the Ce6-P/W NE-treated cells exhibited an evident decrease in viability, as indicated by the Calcein AM signal reduction in the irradiated area (Figure S3). No positive PI signal was detected at these conditions, which suggests that the irradiation dose was not sufficient to trigger necrosis. In contrast, we observed no detectable changes in the viability of Ce6 Es-treated cells under the same irradiation conditions (Figure S3).

**Figure 4:**
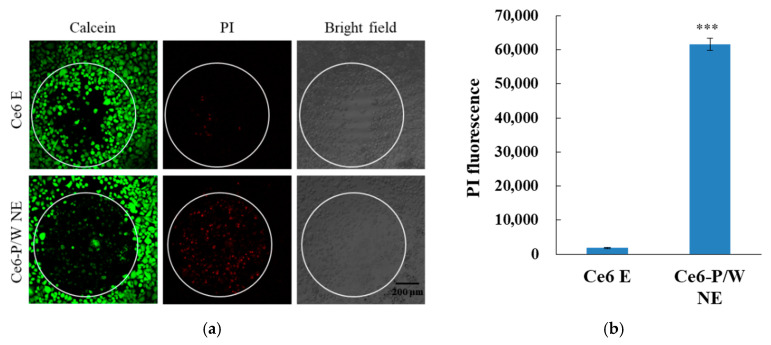
(**a**) Calcein/PI staining and corresponding bright-field images and (**b**) necrosis indicated by PI fluorescence signals of cells treated with Ce6 E or Ce6-P/W NE upon light irradiation (633 nm, 50 mW/cm^2^) in a normoxic condition for 45 s. Ce6-P/W NE concentration, 400 μg/mL. Ce6 concentration, 8 μg/mL. Green color shows living cells, and red color shows dead cells. The white circle indicates the irradiation area. Data were presented as mean value ± standard deviation. After Levene’s test for equality of variances, Student’s t test was employed for the comparison of mean values. n = 3. ***, *p* < 0.001. PI, propidium iodide. Ce6 E, chlorin e6 emulsion. Ce6-P/W NE, chlorin e6-perfluoropolyether (PFPE)/water nanoemulsion.

When, under normoxic conditions, laser irradiation was extended to 45 s for both Ce6 E and Ce6-P/W NE, the treated cells exhibited effective cellular damage, as shown by a reduction in Calcein AM signal intensity and the emergence of a PI signal in the irradiated area. However, the severity of cell damage and necrosis was significantly higher in the Ce6-P/W NE-treated cells, as indicated by the nearly complete suppression of the Calcein AM signal and the presence of numerous necrotic cells, as revealed by positive PI staining (Figure 4a,b).

**Figure 5:**
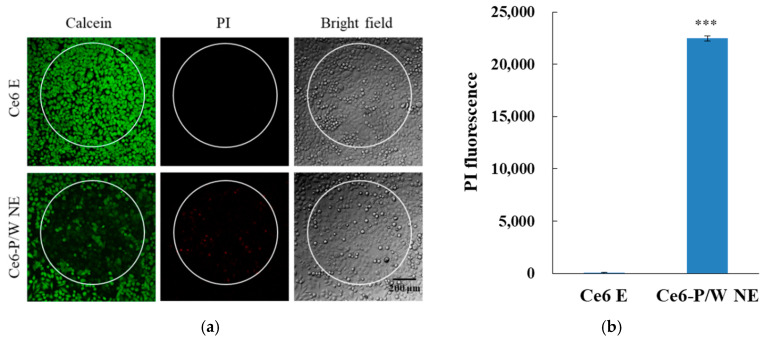
(**a**) Calcein/PI staining and corresponding bright-field images and (**b**) necrosis indicated by PI fluorescence signals of cells treated with Ce6 E or Ce6-P/W NE upon light irradiation (633 nm, 50 mW/cm^2^) in a hypoxic condition for 105 s. Ce6-P/W NE concentration, 400 μg/mL. Ce6 concentration, 8 μg/mL. Green color shows living cells, and red color shows dead cells. The white circle indicates the irradiation area. Data were presented as mean value ± standard deviation. After Levene’s test for equality of variances, Student’s t test was employed for the comparison of mean values. n = 3. ***, *p* < 0.001. PI, propidium iodide. Ce6 E, chlorin e6 emulsion. Ce6-P/W NE, chlorin e6-perfluoropolyether (PFPE)/water nanoemulsion.

In comparison, PDT treatment was significantly less effective under hypoxic conditions. In Ce6 Es-treated cells, we did not find any change in viability, detectable by Calcein and PI staining, even after the increase in irradiation to 105 s, apparently due to ineffective ROS generation under hypoxic conditions. In contrast, the cytotoxicity was evident in the Ce6-P/W NE-treated cells, as shown both by a reduction in the Calcein signal and an increase in the PI signal (Figure 5a,b).

It is noteworthy that the light irradiation power density (50 mW/cm^2^) and irradiation dose (≤5.25 J/cm^2^) in this study are a lot lower than those used on other oxygen-generating Ce6 nanoparticles, which relied on MnO_2_ (150 mW/cm^2^, 90 J/cm^2^ [72] or 100 mW/cm^2^, 30 J/cm^2^ [56]) or H_2_O_2_ (750 mW/cm^2^, 45 J/cm^2^ [73]) decomposition to produce O_2_. This supports the applicability of our PFPE-based nanoformulation (i.e., Ce6-P/W NE) in delivering a large amount of O_2_ to the hypoxic tumor site, consequently improving PDT efficiency.

### 3.5. Cytocompatibility

**Figure 6:**
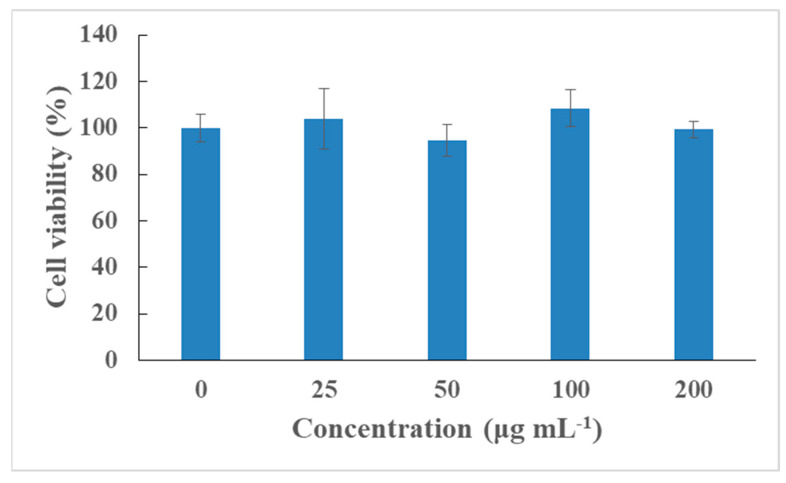
Relative viabilities of PC-3 cells after incubation with various concentrations of Ce6-P/W NE nanodroplets for 24 h. The Ce6 concentrations are 0, 0.5, 1, 2, and 4 μg/mL. Data were presented as mean value ± standard deviation. After Levene’s test for equality of variances, one-way analysis of variance (ANOVA) and Bonferroni multiple comparison test was employed for the comparison of mean values. n = 3. Ce6-P/W NE, chlorin e6-perfluoropolyether (PFPE)/water nanoemulsion.

To further test the cytocompatibility of Ce6-P/W NEs, 24 h incubation was performed with PC-3 cells in the absence of ultrasound or light, and cell viability was measured using the CCK-8 assay. No cytotoxicity could be detected in the tested Ce6-P/W NE concentration range, suggesting sufficient biocompatibility and suitability for biomedical applications (Figure 6) [74].

### 3.6. Tissue-Penetrating Ability of SDT Compared with PDT

**Figure 7:**
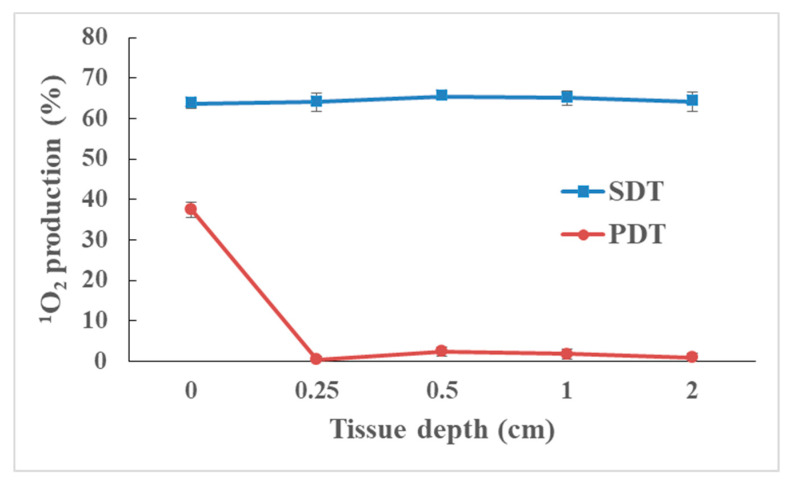
^1^O_2_ production of Ce6-P/W NEs at different tissue depths when irradiated with ultrasound (0.25 W/cm^2^, 2.1 MHz, 1 min, SDT) or white light (60 lumens, 1 min, PDT). Ce6-P/W NE concentration, 400 μg/mL. Ce6 concentration, 8 μg/mL. Data were presented as mean value ± standard deviation. After Levene’s test for equality of variances, one-way analysis of variance (ANOVA) and Bonferroni multiple comparison test was employed for the comparison of mean values. n = 3. The control group receiving no light showed ^1^O_2_ production of (1.026 ± 0.939)%. ^1^O_2_, singlet oxygen. Ce6-P/W NE, chlorin e6-perfluoropolyether (PFPE)/water nanoemulsion. SDT, sonodynamic therapy. PDT, photodynamic therapy.

We investigated the efficiency of ROS generation through various depths of a tissue. Compared with light activation (Figure 7; see also Figure S4 for another Ce6-P/W NE concentration), ^1^O_2_ generation by ultrasound was equally effective throughout the studied tissue depth range (Figure 7). We found no attenuation even for the maximum studied 2 cm depth, which included layers of skin, fat, and muscle. The results here highlight the massive advantage of SDT over PDT for tissue penetration, with a potential to eradicate deep-seated tumors. Consistently, the efficacy of SDT treatment was reported to remain significantly higher than PDT with the increasing guinea pig skin thickness [75]. However, PDT is an intriguing modality for superficial and endoscopically reachable tumors with high spatial selectivity, approaching single-cell level. Such precision is highly valuable for the treatment of tumors located in the brain, as well as in proximity to vital organs, vessels, and glands. Thus, a combination of SDT and PDT serves as a promising synergic modality for cancer treatment, allowing us to target tumors at various depths in the body and meet specific spatial precision requirements. The tumor-targeting effect based on the current nanoplatform may be realized via the following three aspects: (1) Because it has a relatively small droplet size (~150.1 nm) and uniform size distribution, the nano-construct is likely to facilitate effective accumulation of Ce6-P/W NEs into the tumor tissues by passive targeting through the EPR effect in prospective in vivo applications. (2) The spatiotemporal tumor-targeting effect may be achieved by the control of external stimuli including ultrasound and light. The sensitizer Ce6 only generates ROS and damages cells within the specific area and time durations irradiated, as suggested by Figure 3, Figure 4, Figure 5 and Figure 6. (3) Termed as the nanomaterial-induced endothelial leakiness (NanoEL) effect, this phenomenon features the disruption of cell–cell contacts and creation of a temporal gap that enhances the paracellular transportation of molecules across the vascular barrier [76]. The NanoEL effect was categorized into Type I and II. Type I (direct) refers to endothelial leakiness caused by the direct interaction of the nanomaterials with the endothelial cellular components, while Type II (indirect) refers to leakiness resulting from secondary events of nanomaterials’ interactions with the endothelial cells or endothelium [77]. With stimulation by ultrasound and light, our nano-construct may efficiently permeate the endothelial cells at the tumor site via Type II NanoEL from the oxidative stress on the endothelial cells [78]. The NanoEL effect might also be improved by the employment of ultrasound or light [79,80]. In future work, the tumor specificity can be further improved by conjugation of the nanoformulation to tumor-targeting moieties such as folic acid and cRGD. Moreover, further research on the dual action of SDT and PDT effects and in vivo studies using animal models remains to be explored to demonstrate the therapeutic effects. It is likely that the stimulation of both ultrasound and light could improve the therapeutic outcome, as is intensively reported previously [26,31,37,38,39,40].

## 4. Conclusions

In summary, we propose a unified concept that includes a highly potent multifunctional nanoformulation Ce6-P/W NE to affect PDT and SDT under hypoxic conditions. This nanoformulation contains an FDA approved photosensitizer and a uniquely stable high loading efficiency oxygen carrier, which produces no measurable side effects of cytotoxicity. Moreover, Ce6-P/W NEs could be specifically activated in tumors via light and/or ultrasound to produce ROS and eradicate tumors, even in a hypoxic environment, which has been a long-standing translational challenge in this field. The combination of deep tissue penetration of ultrasound and the high spatial precision of light activation for selective treatment of single cells opens up new avenues for therapeutic applications.

In conclusion, the Ce6-P/W NEs-based unified nanoplatform bridges the advantages and eliminates the shortcomings of current PDT and SDT, further advancing ROS-induced cancer therapies.

## Supplementary Materials

The following are available online at https://www.mdpi.com/2079-4991/10/10/2058/s1, Figure S1: Droplet diameter change of the Ce6-P/W NEs in phosphate buffered saline throughout storage at 37 °C for 24 h, Figure S2: ^1^O_2_ production of Ce6 Es and Ce6-P/W NEs under ultrasonic irradiation in normoxic condition at (**a**) 1 mg/mL or (**b**) 0.5 mg/mL Ce6-P/W NE, Figure S3: Calcein staining and corresponding bright-field images of PC-3 cells treated with Ce6 Es or Ce6-P/W NEs upon light irradiation for 30 s in normoxic condition, Figure S4: ^1^O_2_ production of Ce6-P/W NEs at 200 μg/mL at different tissue depth when irradiated with white light.
