# Supplementary material for: Perfluoropolyether Nanoemulsion Encapsulating Chlorin e6 for Sonodynamic and Photodynamic Therapy of Hypoxic Tumor
Source: Nanomaterials (Basel). 2020 Oct 19;10(10):2058. doi: 10.3390/nano10102058 (PMC7603101; doi:10.3390/nano10102058)
Supplement: Supplementary file 1 [file nanomaterials-10-02058-s001.pdf]

nanomaterials

Supplementary Materials

Perfluoropolyether Nanoemulsion Encapsulating Chlorin e6 for Sonodynamic and Photodynamic Therapy of Hypoxic Tumor

Liang Hong [1], Artem M. Pliss [2], Ye Zhan [3], Wenhan Zheng [3], Jun Xia [3], Liwei Liu [1,*], Junle Qu [1,*] and Paras N. Prasad [2,*]

[1] Key Laboratory of Optoelectronic Devices and Systems of Ministry of Education and Guangdong Province, College of Optoelectronic Engineering, Shenzhen University, Shenzhen 518060, China; hongliang0702@126.com

[2] Institute for Lasers, Photonics and Biophotonics, University at Buffalo, State University of New York, Buffalo, New York, NY 14260, USA; ampliss@buffalo.edu

[3] Department of Biomedical Engineering, University at Buffalo, State University of New York, Buffalo, New York, NY 14260, USA; yezhan@buffalo.edu (Y.Z.); wzheng26@buffalo.edu (W.Z.); junxia@buffalo.edu (J.X.)

* Correspondence: liulw@szu.edu.cn (L.L.); jlqu@szu.edu.cn (J.Q.); pnprasad@buffalo.edu (P.N.P.)

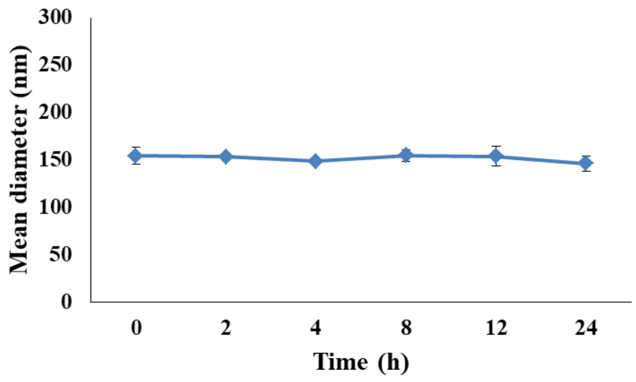

Figure S1. Droplet diameter change of the Ce6-P/W NEs in phosphate buffered saline throughout storage at 37 °C for 24 h. Ce6 concentration, 16 μg/mL. Data were presented as mean value ± standard deviation. After Levene's test for equality of variances, one-way analysis of variance (ANOVA) and Bonferroni multiple comparison test was employed for the comparison of mean values. n = 3. Ce6-P/W NE, chlorin e6-perfluoropolyether (PFPE)/water nanoemulsion.

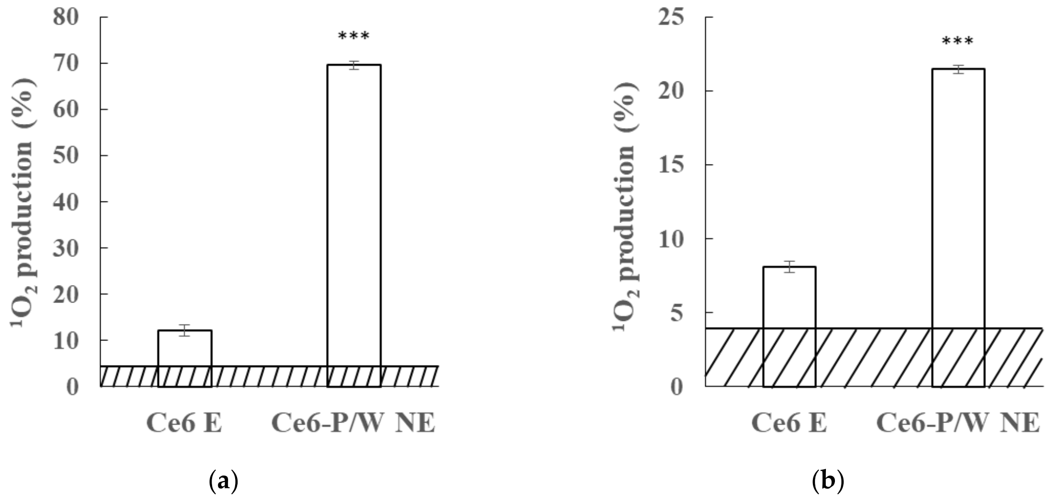

Figure S2. $^1O_2$ production of Ce6 Es and Ce6-P/W NEs under ultrasonic irradiation (0.25 W/cm^2, 2.1 MHz, 1 min) in normoxic condition at (**a**) 1 mg/mL (Ce6 concentration, 20 μg/mL) or (**b**) 0.5 mg/mL (Ce6 concentration, 10 μg/mL) Ce6-P/W NE. Data were presented as mean value ± standard deviation. After Levene's test for equality of variances, one-way analysis of variance (ANOVA) and Bonferroni multiple comparison test was employed for the comparison of mean values. n = 3. ***, $p < 0.001$. The shadow represents background signals detected by control (*i.e.*, water containing only DPBF) groups (2.9922 ± 0.8720%; 4.1107 ± 0.4619%). $^1O_2$, singlet oxygen. Ce6 E, chlorin e6 emulsion. Ce6-P/W NE, chlorin e6-perfluoropolyether (PFPE)/water nanoemulsion. DPBF, 1,3-diphenylisobenzofuran.

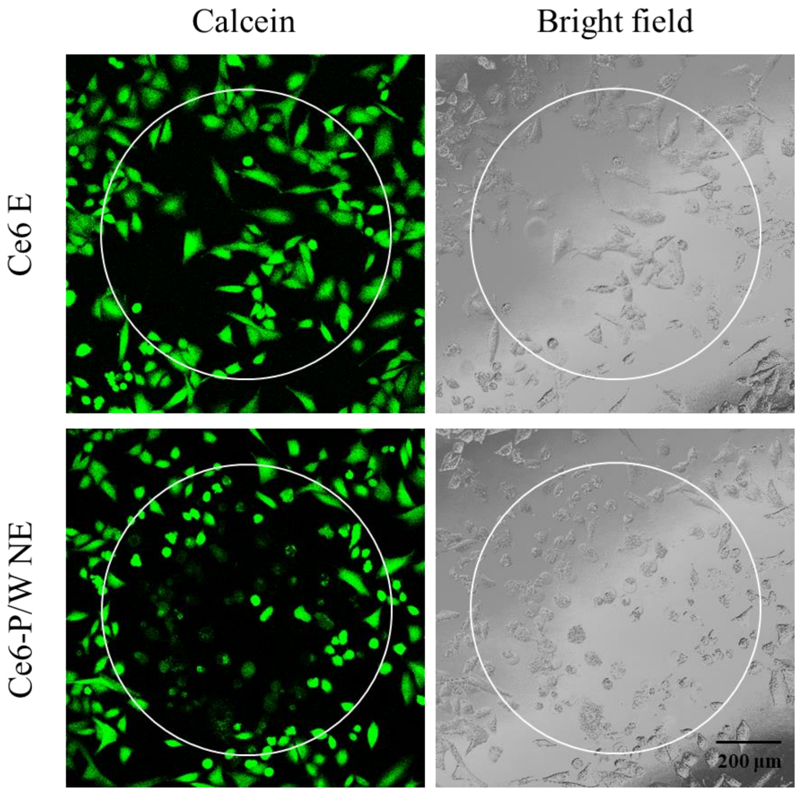

Figure S3. Calcein staining and corresponding bright-field images of PC-3 cells treated with Ce6 Es or Ce6-P/W NEs upon light irradiation (633 nm, 50 mW/cm^2) for 30 s in normoxic condition. Ce6-P/W NE concentration, 400 μg/mL. Ce6 concentration, 8 μg/mL. Green color shows living cells. The white circle indicates the irradiation area. Ce6 E, chlorin e6 emulsion. Ce6-P/W NE, chlorin e6-perfluoropolyether (PFPE)/water nanoemulsion.

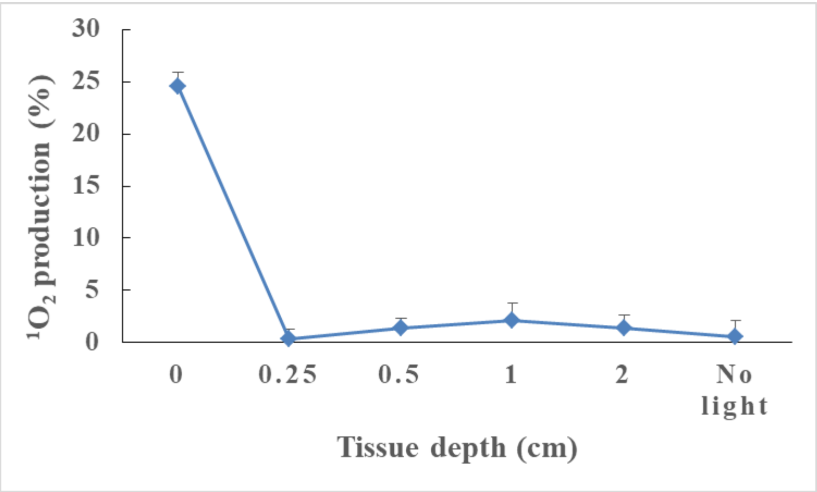

Figure S4. $^1O_2$ production of Ce6-P/W NEs at 200 μg/mL (Ce6 concentration, 4 μg/mL) at different tissue depth when irradiated with white light (60 lumens, 1 min). Data were presented as mean value ± standard deviation. After Levene's test for equality of variances, one-way analysis of variance (ANOVA) and Bonferroni multiple comparison test was employed for the comparison of mean values. n = 3. $^1O_2$, singlet oxygen. Ce6-P/W NE, chlorin e6-perfluoropolyether (PFPE)/water nanoemulsion.

© 2020 by the authors. Submitted for possible open access publication under the terms and conditions of the Creative Commons Attribution (CC BY) license (http://creativecommons.org/licenses/by/4.0/).
